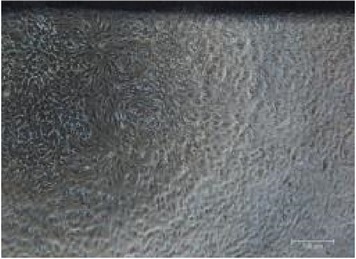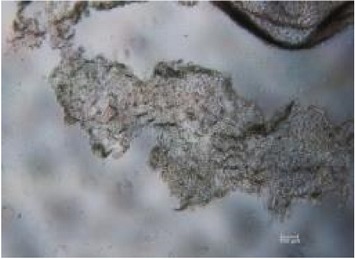# Efficacy Study of Broken Rice Maltodextrin in *In Vitro* Wound Healing Assay

**DOI:** 10.1155/2015/687694

**Published:** 2015-09-08

**Authors:** Zahiah Mohamed Amin, Soo Peng Koh, Swee Keong Yeap, Nur Syazwani Abdul Hamid, Chin Ping Tan, Kamariah Long

**Affiliations:** ^1^Department of Food Technology, Faculty of Food Science and Technology, Universiti Putra Malaysia, 43400 Serdang, Selangor, Malaysia; ^2^Biotechnology Research Center, Malaysian Agricultural Research & Development Institute (MARDI) Headquarters, P.O. Box 12301, 50774 Serdang, Selangor, Malaysia; ^3^Institute of Bioscience, Universiti Putra Malaysia, 43400 Serdang, Selangor, Malaysia

## Abstract

Maltodextrins that contain both simple sugars and polymers of saccharides have been widely used as ingredients in food products and pharmaceutical delivery systems. To date, no much work has been reported on the applications of maltodextrin from broken rice (RB) sources. Therefore, the objective of this work was to investigate the* in vitro* wound healing efficacy of RB maltodextrin at different conditions. Wounds treated with lower dextrose equivalent (DE) range (DE 10–14) of maltodextrins at a concentration of 10% obtained from RB were found to be able to heal the wounds significantly faster (*p* < 0.01) than maltodextrin with higher DE ranges (DE 15–19 and DE 20–24) and concentrations of 5% and 20%. The findings from both BrdU and MTT assay further confirmed its wound healing properties as the NIH 3T3 fibroblast wounded cells were able to proliferate without causing cytotoxic effect when wounded cell was treated with maltodextrin. All these findings indicated that the RB maltodextrin could perform better than the commercial maltodextrin at the same DE range. This study showed that RB maltodextrins had better functionality properties than other maltodextrin sources and played a beneficial role in wound healing application.

## 1. Introduction

Rice constitutes the world's principal source of food. For example, it is the major source of dietary energy and protein for 80% of the population in Southeast Asia [1]. About 14% of broken rice (RB) is generated during rice milling processing leading to a direct economic loss to millers [2]. In the past, RB was used in beer making [3], and now, RB is used for commercial broilers to reduce the cost of poultry production and sparing maize for other uses [4]. This low valued by-product from rice milling industry should be used for applications with better economic returns [5]. Rice is rich in starch, containing about 88% on average [2]. According to a study conducted by Guenoun et al. [6], broken rice constitutes 82.31% of starch yield. This rich in starch source is an ideal source to produce a high quality grade of maltodextrin for the application in food and pharmaceutical industries.

Enzymatic modification of starch involves starch hydrolysis using amylolytic enzymes to break the polymer of starch molecules into a lower molecular-weight called maltodextrin, or dextrin, which is widely used in food and pharmaceutical industries [7]. To date, the common sources of maltodextrin production include corn, pea, potato, wheat, sorghum, maize, and tapioca [8]. In general, maltodextrins are characterized by dextrose equivalent (DE) value, which expresses the level of starch conversion. The DE value, describing the total reducing sugar content of a material, is expressed as percent of dextrose in dry basis [9]. Maltodextrin has been previously reported as a potential wound healing agent by promoting the proliferation of fibroblast cells [10]. Low DE maltodextrin is more preferable as a wound healing agent due to the presence of higher content of long oligomer chains [11]. As described in the U.S. Patent number 0,018,955, maltodextrin with a low DE value is capable of forming a film, which is intimately adhered to the underlying granulation tissue. Low DE maltodextrin is semipermeable to gas and fluids and thus provides an ideal protective cover to reduce the loss of fluid and plasma and the invasion of pathogenic microorganisms [12]. Moreover, a gradual release of small amount of glucose content in low DE maltodextrin is particularly effective to provide topical nutrition to the wound site, creating a natural wound healing environment [12].

Wound healing process consists of a series of recovery steps: (a) injured tissue is repaired; (b) specialized tissue is regenerated, and (c) new tissue is reorganized [13]. When cells are injured or killed from a wound, a wound healing step is required to resuscitate the injured cells and produce new cells to replace the dead cells. The healing process requires the reversal of cytotoxicity, the suppression of inflammation, and the stimulation of cellular viability and proliferation [14]. Diseases such as diabetes, immunocompromised, ischemia, and other conditions like malnourishments, ageing, local infections, and local tissue damaged wounds could cause a delay in the healing process [15]. Such conditions certainly require the use of healing agents to facilitate the wound healing process. One of the major problems with many known film forming agents is that they are rarely capable of enhancing the wound healing process. Therefore, in the wound of any substantial size, skin grafting will always be required [16]. Most published wound healing studies focused on microfluidic wound healing treatment [17], wound healing comparative studies [18], radiation therapy treatment [19], or topical ointment treatment [20].

Up to date, there is no information on the use of RB maltodextrin as a wound healing agent, reported. Thus, RB maltodextrin with different DE groups was produced and subjected to an* in vitro* wound healing and proliferation assay on NIH 3T3 cell line. The main objective of this study is to examine the wound healing efficiency of RB maltodextrin using an* in vitro* model on NIH 3T3 fibroblast cells and at the same time, comparison of its wound recovery rate with a commercial maltodextrin will be carried out to confirm the quality of maltodextrin produced from RB sources.

## 2. Materials and Methods

### 2.1. Materials

Mature but unripened RB (blends of local varieties, MR 219 and MRR 220) were purchased from the local market (Serdang, Selangor). All starches were prepared at the laboratory scale [21, 22]. RB maltodextrin of different DE group (DE 10–14, DE 15–19, and DE 20–24) was supplied by MARDI (Serdang). A 10% of commercial (COM) maltodextrin (DE 10–14) was used as a reference as it was known to produce high quality maltodextrin [22]. Multidex, a known commercial wound dressing agent containing maltodextrin, was used as a comparison purpose (DE 15–19).

### 2.2. Culture of Cell Lines

NIH 3T3 cell line was obtained from ATCC, USA, and cultured in RPMI 1640 medium supplemented with 1% penicillin-streptomycin and 10% fetal bovine serum (FBS) in a humidified incubator with 5% CO_2_. Cell line was detached from the culture flasks using a trypsin-EDTA solution (0.25–0.025%) and resuspended as a single cell suspension in RPMI 1640 culture medium.

### 2.3. *In Vitro* Wound Scratch Assay and Microscopy Evaluation

NIH 3T3 cells were seeded in a tissue culture 6-well plate at an initial density of 2.4 × 10^5^ cells/cm^2^ overnight. A micropipette tip was used to create a wound in the monolayer by scraping. A total of 10 (%, w/v) RB maltodextrin were added in each treatment well with or without an addition of 100 ppm of various additives including* aloe vera*, curcumin, hydroxyproline, ascorbic acid, L-arginine, lactic acid, and kojic acid, which were added separately to each well. Another two wells were treated with Multidex and media only (control), respectively. Wound closure was observed by phase-contrast microscopy (NIKON, Japan) and digital images were taken at the interval time of 3 h up to 24 h.

### 2.4. Determination of NIH 3T3 Cell Viability* via* Trypan Blue Cell Count Assay

Trypan blue cell count was carried out to identify the amount of viable cells present in each sample. After 24 h incubation period, harvested cell suspension (10 *μ*L) was added with equal volume of 0.4% trypan blue stain. Hemocytometer was used for cell counting under inverted light microscope (NIKON, Japan). Viable cells are those excluded from the stain.

### 2.5. 5-Bromo-2′-deoxyuridine (BrdU) ELISA Cell Proliferation Assay

Maltodextrin treated and untreated NIH 3T3 cell proliferation was measured using the Bromodeoxyuridine (BrdU) Cell Proliferation Kit (Merck, USA). The cells were seeded in a 96-well plate at a concentration of 0.8 × 10^5^ cells/mL overnight. A total of 10% RB maltodextrin were added separately with or without the addition of 100 ppm of various additives, including* aloe vera*, curcumin, and hydroxyproline and incubated for 24, 48, and 72 h, respectively, at 37°C and 5% CO_2_. Another two wells were treated with Multidex and media only to serve as untreated control. After the corresponding period, BrdU label was added into all wells and incubated for an additional 24 h. At the respected incubation hours, the cells were fixed and incubated at 4°C for approximately 30 min. After that, the plates were washed twice, added with 100 *μ*L detector antibodies into each well, and incubated for 1 h. Then, 100 *μ*L of goat anti-mouse Ig G-HRP conjugated was added and incubated for 30 min. Then, the plates were incubated with 100 *μ*L of 3, 3′, 5, 5′-Tetramethylbenzidine (TMB) substrate for another 30 min. Finally, 100 *μ*L of stop solution (sulfuric acid) was added and the absorbance was measured at 450 nm, using an ELISA microplate reader (Biotech Instruments, USA).

### 2.6. 3-(4,5-Dimethylthiazol-2-yl)-2,5-diphenyl Tetrazolium Bromide (MTT) Cell Viability Assay on NIH 3T3 Cells

NIH 3T3 cells were seeded on 96-well microtiter plates overnight. A respective amount of 5% and 10% of RB maltodextrin was added in each treatment well separately. An addition of 100 ppm of various additives including* aloe vera*, curcumin, and hydroxyproline was added separately to each well. Both Multidex and media acted as control. The fibroblast cells treated with various samples were exposed to the culture medium up to 72 h. At each interval time of 24 h, a total of 20 *μ*L/well of MTT solution (Calbhiochem, USA) were added, followed by incubation at 37°C for a period of 4 h in an atmosphere of air with 5% CO_2_. After that, supernatants were removed from the wells and 100 *μ*L/well of dimethyl sulfoxide (DMSO) (Fisher, USA) was added to solubilize formazan. The absorbance was quantified at 570 nm using ELISA microplate reader (Biotech Instruments, USA).

### 2.7. *In Vitro* Red Blood Cell (RBC) Irritation Assay

Approximately 2 mL of RBC obtained from a volunteer (Pusat Kesihatan, UPM) and was washed in PBS in a ratio of 1 : 10, followed by centrifuged for 10 min at 1500 rpm with a controlled temperature of 10°C, and this step was repeated triplicate. The RBC was then diluted with PBS to a 1% concentration. Each 100 *μ*L of RB, COM maltodextrin, and Multidex were loaded into the first well in a 96-well plate separately, followed by 50 *μ*L of PBS loaded from the 2nd to the 12th well. A serial dilution was carried out from the 1st well to the 11th well and the 12th well was treated as the positive control. Then, a total of 50 *μ*L of 1% RBC were added to each well before being incubated at room temperature for 30 min. At the end of incubation, the suspension was centrifuged at 500 rpm at 10°C for 10 min.

### 2.8. Statistical Analysis

Data was statistically analyzed by one-way analysis of variance (SPSS statistics version 16). Significant differences (*p* < 0.01) between means were determined by Duncan's multiple range test.

## 3. Results and Discussion

### 3.1. A Preliminary Wound Healing Comparison Study on RB Maltodextrin with the Addition of 100 ppm of Various Additives

*In vitro* wound healing process starts with the spreading of individual cells at the wound edge and the synthesis of matrix fibrils (e.g., fibronectin), followed by cell migration (translocation) along the fibronectin and cell proliferation process [23].  Table 1 shows the results of a preliminary study of* in vitro* wound healing on RB maltodextrin. In this study, the NIH 3T3 cells were seeded in six-well culture plates to test the wound healing effect of RB maltodextrin with and without 100 ppm of various additives. Overall, this study consisted of three parameters with the aims to examine the wound healing effect of (a) different concentration of additives choice; (b) different concentration of RB maltodextrin; and (c) different DE grades of RB maltodextrin. These factors were studied to identify the optimum condition of RB maltodextrin to assist the wound healing of NIH 3T3 cells.

Various additives including curcumin, hydroxyproline, ascorbic acid, L-arginine, lactic acid, and kojic acid were selected based on previous publications on their significance to promote wound healing properties [24–28].* Aloe vera* is a tropical cactus which has been reported to have therapeutic potential in a variety of soft tissue injuries, medical and cosmetic purposes, and general health [29]. A study carried out by Vera [30] reported that 50.8% improvement in wound closure was observed in mice when treated with topical* aloe vera*. Heggers [31] study confirmed the therapeutic effects of* aloe vera*, whereby it showed the progressive prevention of tissue loss in dermal ischemia caused by burns, frostbite, electrical injury, distal dying flap, and intra-arterial drug abuse in both man and animal models. Therefore, 100 ppm of* aloe vera* was added to examine its proliferation effect on NIH 3T3 wounded cells. According to Schreier et al. [23], the addition of additives (platelet-derived growth factor) was noted to increase the cell migration to the “wounded” area compared to cells in the absence of additives. Furthermore, active additives may promote the process of wound healing by increasing the viability of collagen fibrils and the strength of collagen fibers, either by increasing the circulation or by preventing the cell damage or by promoting the DNA synthesis [23]. Multidex, one of the popular commercial wound dressings, was used as a comparative control while the cells containing only fresh media without any treatment acted as the control.

Additives act as growth factors to promote the tissue repair, migrating cell into the wound site and stimulating cell proliferation [32]. Initially, a study of using different additives concentration effect on the wound closure was conducted to determine its healing capability of NIH 3T3 cells. Based on the results (Table 1(a)), the response obtained from each NIH 3T3 cells treatment exposed to different concentrations of additives was distinguishable (*p* < 0.01) than responses received in the control group. In brief, the use of 50 ppm additives was noted to be insufficient to heal the wounds within 24 h relative to 100 ppm additives.

It is important to identify the concentration at which maltodextrin can perform the best healing capability [33]. Therefore, different concentrations of RB maltodextrin (5%, 10%, and 20%) with the same DE value of 10–14 were conducted (Table 1(b)). Addition of 100 ppm additives to each maltodextrin was studied also in the wound healing process of NIH 3T3 cells. The response obtained from each NIH 3T3 cells after exposure to different concentrations of maltodextrin was distinguishable (*p* < 0.01) than responses received in the control group. In Table 1(b), treatments with either 5% or 20% RB maltodextrin were not able to heal the wounds completely within 24 h. These two concentrations did not show significant improvement (*p* < 0.01) in the percentage of wound closure relative to the control group. The addition of 5% RB maltodextrin into NIH 3T3 wounded cell was found to be too liquefied and could not provide sufficient nutrients to the cells leading to poor cell migration. On the other hand, high concentration of RB maltodextrin (20%) was not suitable to be used in wound healing as it was too concentrated and yield a syrup-like characteristic due to high solid content [9]. These results indicated that high viscosity of RB maltodextrin prevents the absorbance of nutrients to the cells to enhance the proliferation and migration of the cells. Only 10% concentration of RB maltodextrin was capable of achieving 100% recovery within 24 h as shown in Tables 1(b)(ii) and 2. It was also noted that 10% RB maltodextrin alone showed the best wound healing of NIH 3T3 at 12 h (61.13%), followed by 10% RB maltodextrin with 100 ppm curcumin (52.97%), 10% RB maltodextrin with 100 ppm hydroxyproline (51.52%), 10% RB maltodextrin with 100 ppm ascorbic acid (47.78%), 10% RB maltodextrin with 100 ppm L-arginine (47.66%), 10% RB maltodextrin with 100 ppm lactic acid (44.94%), and 10% RB maltodextrin with 100 ppm kojic acid (32.98%). Multidex was found to cause a cytotoxicity rather than wound healing effect on the NIH 3T3, whereby the cells were observed to be unhealthy and unable to migrate and died within 12 h as shown in Table 2. This may be contributed by the presence of preservative in the Multidex [6] since Multidex is formulated for external use only and may not be a suitable agent for* in vitro* wound healing study.

The third parameter of the preliminary study was to investigate the effect of different DE ranges of 10% RB maltodextrin (DE 10–14, DE 15–19, and DE 20–24) on its healing capability of NIH 3T3 cells. Based on the results obtained, RB maltodextrin DE 10–14 (Table 1(c)) performed the best wound healing of NIH 3T3 relative to either RB maltodextrin DE 15–19 or DE 20–24. Although most of the RB maltodextrin DE 15–19 series treatments were able to heal the wounds within 24 h, RB maltodextrin DE 10–14 was found to be able to perform significantly better (*p* < 0.01) in all treatments than RB maltodextrin DE 15–19 group. On the contrary, RB maltodextrin DE 20–24 was noted to be unable to heal the wounds completely even after 24 h and showed significantly the poorest performance (*p* < 0.01) relative to the other RB maltodextrins with different DE groups. Maltodextrin with a lower DE value is preferable to facilitate the exposure of dermatological agents added to improve the healing and facilitates the contact to all areas of the wound [16]. The DE value of maltodextrin affects the viscosity, sugar composition, and the characteristics of the maltodextrin [34]. According to Sun et al. [35], the molecular composition of maltodextrin differs at different DE values. Maltodextrin with a lower DE range possessed higher molecular weight and longer chain of glucose polymers [35]. The presence of short chain sugar molecules in the maltodextrin DE 10–14 provided sufficient nutrient and showed better stimulation and migration of the cells [10]. On the other hand, a higher DE value of maltodextrin (DE 15–19 and 20–24) tends to increase the viscosity and provides a more sugary characteristic, subsequently affects the cell migration, and thus decreases the ability of maltodextrin to diffuse into the cell monolayer [10].

Overall, 10% RB maltodextrin DE 10–14 had shown the best performance in healing NIH 3T3 wounded cells relative to 5%, 20% RB maltodextrin concentrations, and also RB maltodextrin with higher DE value (DE 15–19 and DE 20–24 group). In comparison to various additives effect on the percentage wound closure of NIH 3T3 fibroblast cell, both lactic acid and kojic acid performed significantly the poorest (*p* < 0.01) compared to the other additives. Lactic acid and kojic acid are mostly used in the cosmetic area and may not be very suitable in the wound healing of NIH 3T3 cells as these additives may have the potential to cause irritations if not used at a suitable amount [36]. However, the wounded cells treated with curcumin and hydroxyproline showed significantly higher (*p* < 0.01) healing power compared to other additives. This phenomenon may be caused by their attribution to increase the stimulation of the fibroblast cells proliferation [29]. Curcumin, the active ingredient in the spice, turmeric, has been found to be effective in the skin injury treatment, whereas hydroxyproline, an amino acid, is unique for collagen that helps in the tissue recovery of wound area [27, 37]. The addition of 100 ppm additives did improve the percentage wound closure compared to media alone. However, 10% RB maltodextrin DE 10–14 alone had shown significantly the best recovery rate (*p* < 0.01) in the wound healing compared to the treatment with addition of various additives. The purpose of adding various types of additives was to compare the healing effects of these additives with RB maltodextrin. This finding indicated that 10% RB maltodextrin DE 10–14 was the best wound healing agent and proved its effectiveness to facilitate wound healing process of NIH 3T3 cells.

To further investigate the wound healing capability of other maltodextrin source on the NIH 3T3 wounded cell, a commercial (COM) maltodextrin from cassava source was studied under the same condition. Cassava maltodextrin was selected as it was known of its high quality maltodextrin and widely used in the food and pharmaceutical applications. A previous comparison study between RB and cassava starch by Koh and Long [22] had confirmed their differences in the physicochemical properties, and eventually it will affect their functional property when these starches were hydrolyzed to produce maltodextrin.

**Figure 1:**
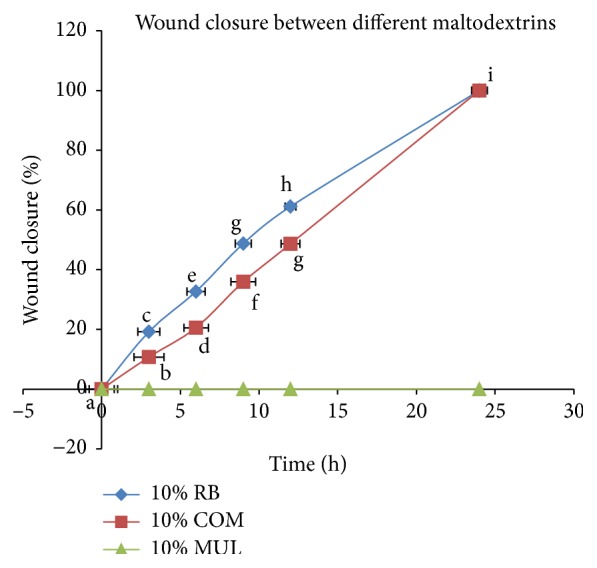
*In vitro* wound scratch assay on NIH 3T3 fibroblast cell using different sources of maltodextrins. The values were the means ± SE of ten independent experiments. The differences between the control group and treated group were determined by one-way ANOVA (^a^ *p* < 0.01). RB: broken rice maltodextrin; COM: commercial maltodextrin; MUL: Multidex.

Figure 1 shows the wound healing performance of 10% RB maltodextrin, 10% COM and 10% Multidex. Based on the graph, it showed that the percentage wound closure of NIH 3T3 wounded cell when treated with RB maltodextrin was higher than COM maltodextrins. Although RB and COM maltodextrins were under the same DE range, the time taken for each cell migration in the wound closure study was found to vary, significantly depending on its maltodextrin source. This is proportional to the earlier described statement; maltodextrin with the same DE ranges from different starchy sources has different functional and physiochemical properties, which is highly dependent on the starch molecular structure itself [34, 35]. As reported herein, RB maltodextrin could significantly stimulate the proliferation of NIH 3T3 cells better than COM maltodextrin. One of the possible pros reason to explain the less efficient performance of COM maltodextrin in the wound healing relative to RB maltodextrins was due to its molecule size. According to Koh and Long [22], cassava starch possessed the largest molecule size compared to RB starches as shown by a micrograph study. The larger molecule size of COM maltodextrin may have led to poor nutrient supplement to the NIH 3T3 cells and thus slowed down the rate of cell migration. Overall, it was clearly shown that RB maltodextrins had positive influence on its characteristics in the wound healing functional property, which substantiates it as a wound healing agent. In this study, we have proven that RB maltodextrins have performed significantly better compared to the COM maltodextrin as wound healing agent.

An occlusive dressing may facilitate a moist wound environment and retains the wound fluid and its various components; however, it also keep oxygen away from the tissues at the same time. Oxygen plays an important role in the collagen synthesis [38]. One of the important functions of maltodextrin in the wound healing application is the formation of a film, which is intimately adhered to the underlying granulation tissue. This film is semipermeable to gas and fluids, providing an ideal protective cover to reduce the loss of fluid and plasma and invasion by pathogenic bacteria [12]. This finding indicated that our newly produced RB maltodextrins not only enhance the speed recovery rate of wound healing but also are capable of forming a thin protective layer over the wound that subsequently allowed the exchange of oxygen and retaining the required sugars (from maltodextrin) as a nutritious source to the wounded cells for the proliferation of new cells. Even though our study was conducted using* in vitro* model on NIH 3T3 fibroblast cell, Heng's [38] study had supported our findings and they claimed that sugar dressings (maltodextrin) tested on wounded dogs and cats in a veterinary clinical study capably drew macrophages into the wound and accelerated sloughing of necrotic tissue which enhanced the recovery rate of wound healing process. In addition, their study also reported that the supply of simple sugar to the wounded site acted as a local nutrient source which decreased the incidence of inflammatory edema and subsequently sped up the cell granulation and epithelialization process.

### 3.2. Determination of NIH 3T3 Cell Viability* via* Trypan Blue Cell Count Assay

A well-recognized essential requirement of most biological investigations using cellular preparations is the assertion of cell viability [39]. The most widely applied criterion for investigation of cell permeability is the exclusion by cell of dyes with higher molecular weights (vital stains) such as trypan blue [39]. Trypan blue is a vital dye in which its chromophore is negatively charged and does not interact with the cells unless the membrane is damaged [40]. Therefore, cells which exclude the dye are considered to be viable.

**Figure 2:**
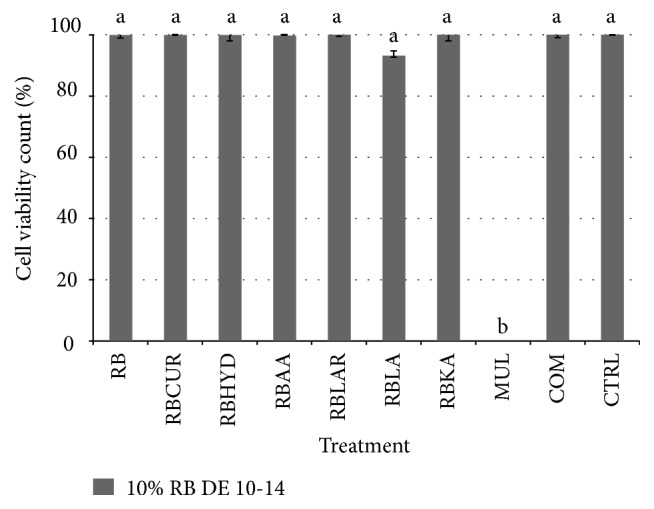
The percentage of viable cells treated with 10% RB maltodextrin DE 10–14 with 100 ppm of various additives at 24 h incubation period. The values were the means ± SE of ten independent experiments. The differences between the control group and treated group were determined by one-way ANOVA (^a^ *p* < 0.01). RB; broken rice maltodextrin; CUR: curcumin; HYD: hydroxyproline; AA: ascorbic acid; LAR: L-arginine; LA: lactic acid; KA: kojic acid; MUL: Multidex; COM: commercial maltodextrin; CTRL: control.

Figure 2 represents the trypan blue cell count assay of NIH 3T3 cell treated with RB maltodextrin, to evaluate the percentage of viable cells during* in vitro* wound healing study. In general, all trypan blue cell count results were noted to tally with the percentage of wound healing findings. It was found that those treatments had shown an improvement in the wound closure possessed higher percentage of cell viability.

Based on the cell counts findings, the cell viability of all maltodextrin treatment groups achieved 100% viability rate, except for RBLA treatment, which only showed the cell viability at the rate of 93%. This phenomenon indicated that the presence of lactic acid had an effect on the cell viability of NIH 3T3 fibroblast cell. Although RB maltodextrin alone was shown to be able to heal the wounds better than the same treatment with addition of other additives, most of the cells treated with additives were able to maintain 100% viability. In all of the results presented, it was found that Multidex, a commercial wound dressing, showed zero percentage of cell viability. This finding indicated that the presence of Multidex in the NIH 3T3 cells most probably had caused substantial cell damage over time after treatment, leading to the increase in the cell death [6]. Generally, the trypan blue cell count assay had supported the findings of wound healing capability of RB maltodextrins with DE value of 10–14 as confirmed in the percentage of wound closure observed in the NIH 3T3 wounded cells.

### 3.3. BrdU ELISA Cell Proliferation Assay

**Figure 3:**
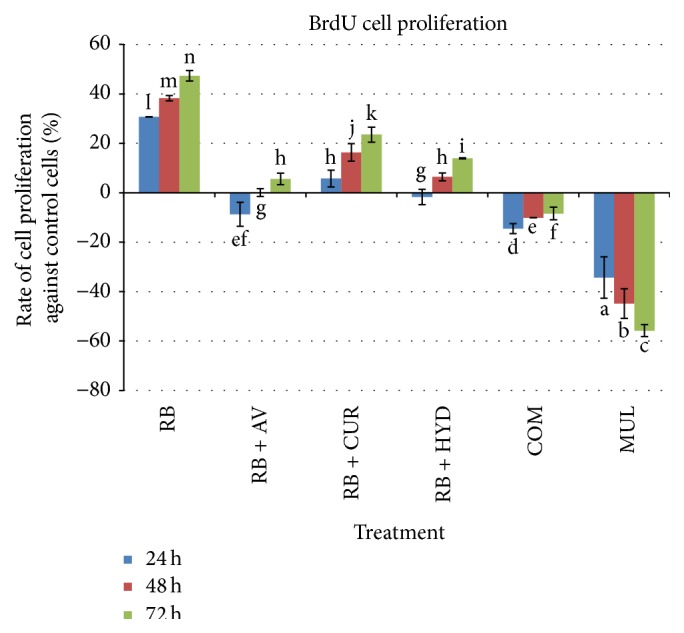
BrdU cell proliferation of NIH 3T3 cells treated with 10% RB maltodextrin with 100 ppm of various additives compared to the cell proliferation of the controlled NIH 3T3 cells at 24, 48, and 72 h incubation period. The values were the means ± SE of seven independent experiments. The differences between the control group and treated group were determined by one-way ANOVA (^a^ *p* < 0.01). RB: broken rice maltodextrin; AV:* aloe vera* extract; CUR: curcumin; HYD: hydroxyproline; COM: commercial maltodextrin; MUL: Multidex.

Freshney [41] has shown that proliferation of fibroblast can be observed by BrdU incorporation. Thus, BrdU ELISA cell proliferation assay was used to evaluate the proliferative effect of maltodextrin on NIH 3T3 fibroblast cell* in vitro*. Cultured cells that entered the log growth phase are pulsed to be labelled with the nonradioactive BrdU [42]. The proliferation of NIH 3T3 cells treated either with 10% RB maltodextrin with or without additives compared to the controlled NIH 3T3 cells that are cultured with media only is shown in Figure 3. Generally, the proliferation rate of NIH 3T3 cells was noted to increase gradually across time comparing to the controlled cells, except for COM and Multidex treatment, whereby the percentage of cell proliferation decreased significantly (*p* < 0.01) from 24 to 72 h.

Overall, all treated NIH 3T3 cells (except COM and Multidex) achieved the highest percentage of cell proliferation after 72 h of incubation. Increase in the percentage of cell proliferation may be attributed to the stimulation by maltodextrin to promote the propagation of fibroblast cells. Cells treated with 10% RB maltodextrin with or without additives showed a higher percentage of cell proliferation than the control group. In general, the highest percentage of cell proliferation at 72 h was RB maltodextrin, which was 47.33% higher in proliferation compared to the control group, followed by RB maltodextrin with 100 ppm curcumin (23.53%), RB maltodextrin with 100 ppm hydroxyproline (13.91%), and RB maltodextrin with 100 ppm* aloe vera* extract (5.60%). However, cells treated with COM and Multidex maltodextrin possessed a lower proliferation rate compared to the control group across time. Overall, our newly developed RB based maltodextrin alone had proven to have higher cell proliferation rate, which was also confirmed through the findings that indicated its better functional property in the wound healing application. This effect may be contributed by the presence of glucose in maltodextrin, which supplied energy to the metabolism and proliferation of the cell [10]. Thus, this finding confirmed that NIH 3T3 wounded cells that were treated with nutrient rich maltodextrin improved the proliferation of cells; therefore it showed better performance in the proliferation of cells as opposed to the untreated cells.

### 3.4. MTT Cell Viability Assay on NIH 3T3 Cells

**Figure 4:**
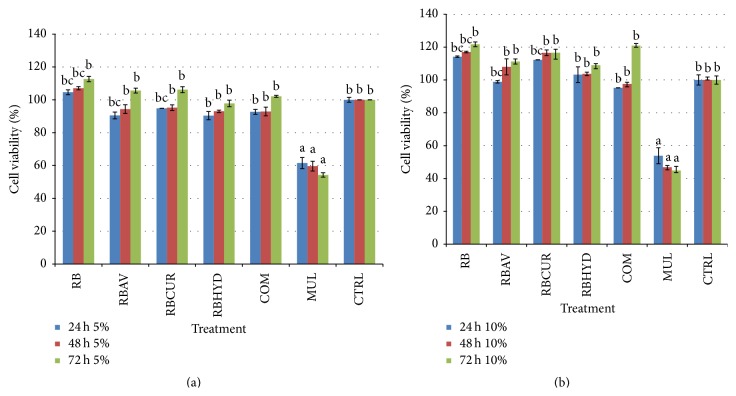
MTT cytotoxic assay of NIH 3T3 cell at 24, 48, and 72 h incubation period treated with (a) 5% RB maltodextrin with 100 ppm of various additives and (b) 10% RB maltodextrin with 100 ppm of various additives. The values were the means ± SE of seven independent experiments. The differences between the control group and treated group were determined by one-way ANOVA (^a^ *p* < 0.01). RB: broken rice maltodextrin; AV:* aloe vera* extract; CUR: curcumin; HYD: hydroxyproline; COM: commercial maltodextrin; MUL: Multidex.

MTT assay was performed to determine the cell viability of NIH 3T3 cell after being treated with maltodextrin. This test involves the conversion of tetrazolium salt, 3-(4,5-dimethylthiazol-2-yl)-2, 5 diphenyl tetrazolium bromide (MTT) to an insoluble formazan product, which is quantitated by spectrophotometric method [27].  Figure 4 represents the cell viability of NIH 3T3 cells treated with RB maltodextrin at (a) 5% and (b) 10% concentrations, with or without the addition of 100 ppm additives, respectively. Generally, the cell viability of RB maltodextrins at 5% and 10% concentration was observed increased significantly (*p* < 0.01) with the incubation time. The increase in the cell viability percentage had indicated that the cells were still viable even after culture up to 72 h. In comparison to 5% maltodextrin, the cell viability of NIH 3T3 cells treated with 10% RB maltodextrins was shown significantly (*p* < 0.01) to be higher. Similar to BrdU proliferation assay's findings, MTT cell viability assay also showed a significant decrease (*p* < 0.01) in the percentage of cell viability in Multidex treated group, as observed at 24, 48, and 72 h. Toxicity appeared in the cells treated with either 5 or 10% Multidex which had caused a decrease in the percentage of cell viability from 61.46% to 54.22% for 5% Multidex and 53.85% to 44.83% for 10% Multidex after 72 h of incubation, respectively.

Briefly, the NIH 3T3 cell treated with 10% RB maltodextrin emerged to have the highest percentage of cell viability after 72 h (121.70%), followed by COM maltodextrin (120.96%), RB maltodextrin with 100 ppm curcumin (116.60%), RB maltodextrin with 100 ppm* aloe vera* extract (111.21%), and RB maltodextrin with 100 ppm hydroxyproline (108.94%). Similar to the findings as reported in the BrdU cell proliferation assay, RB maltodextrin alone possessed higher cell viability relative to other treatments.

### 3.5. *In Vitro* RBC Irritation Assay

The criticism of the classical* in vivo* methods to predict skin or ocular irritation brings up the development of a number of replacement* in vitro* methods, which have been recently reviewed to reduce the use of animal for this purpose [37].* In vitro* studies using RBC are an alternative technique to the* in vivo* eye irritation test since it is an inexpensive and rapid and provides reliable results with good reproducibility method that reduces and even avoids the use of experimental animals for this kind of test [31]. When the RBC is in contact and in circular shape, it indicates that the tested sample does not cause irritation to the cell and thus the sample is safe to be used on human skin. If the RBC lyse, this indicates that the sample at the tested concentration had irritation effects.

**Figure 5:**
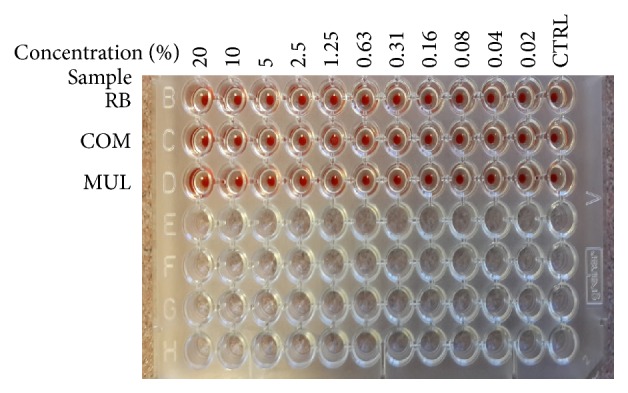
*In vitro* RBC hemolysis assay with various maltodextrins at 30 min incubation period. RB: broken rice maltodextrin; COM: commercial maltodextrin; MUL: Multidex.

Figure 5 shows the RBC hemolysis assay results when tested with various concentrations of RB maltodextrin, COM maltodextrin, and Multidex. Wells consisting either PBS or RBC were served as positive control. When the maltodextrin treatments were added to the erythrocyte suspension in aqueous medium, they will be distributed between the erythrocyte membrane and the solution by absorption first until the equilibrium is reached [43]. Hemolysis probably begins when the erythrocyte membranes are saturated with the treatment molecules [43]. Based on the results, all treatments had no lysis effect on the hemolytic activity. This finding proven that maltodextrin is safe to be used topically and would not cause any irritation to the human skin. Multidex also did not cause the RBC to lyse although it caused cell death in the* in vitro* wound healing. The presence of preservative in Multidex reduced the cell viability on NIH 3T3 cells, which were sensitive to the cells. This finding showed that the preservatives in Multidex are safe to use on the human skin but not safe to be consumed orally or injected into the skin. In conclusion, RB maltodextrin was confirmed to be safe and proven to have no hemolytic effects even at a high concentration treatment.

## 4. Conclusion

RB maltodextrins with low DE group (DE 10–14) showed better improvement of the wound closure compared to high DE group (DE 15–19 and DE 20–24) as proven in the* in vitro* wound healing model. Interestingly, RB maltodextrin with low DE value alone treatment had exhibited the best recovery rate in wound healing treatment by an evidence shown in the wound scratch assay, trypan blue, MTT cytotoxic, and BrdU cell proliferation assay compared with those treatment containing the addition of additives. When compared to the COM maltodextrin, RB maltodextrins exhibited higher healing efficiency as shown in the wound healing, MTT cell viability, and BrdU cell proliferation assays. More importantly, RB maltodextrins also did not induce irritation effect in the RBC assay. In conclusion, RB maltodextrin has successfully shown a positive influence to speed up* in vitro* wound closure and play a beneficial role in wound healing.

## Figures and Tables

**Table:** (a)

Time, h	RB	RBCUR	RBHYD	RBAA	RBARG	RBLA	RBKA	MUL	CTRL
(i) The percentage of wound closure of NIH 3T3 cell treated with 10% RB maltodextrin (DE 10–14) with 50 ppm of various additives									
0					0.00 ± 0.00_a_^A^				
3	5.69 ± 3.05_a_^A^	9.23 ± 0.00_b_^AB^	6.43 ± 1.84_a_^A^	6.16 ± 3.37_a_^A^	8.64 ± 3.92_ab_^AB^	5.81 ± 3.80_ab_^A^	4.82 ± 4.43_a_^A^	0.00 ± 0.00_a_^A^	12.04 ± 0.53_b_^D^
6	15.72 ± 5.95_b_^A^	15.38 ± 0.00_b_^A^	18.21 ± 8.87_ab_^A^	13.43 ± 4.89_b_^A^	18.06 ± 5.25_bc_^A^	15.48 ± 3.92_b_^A^	10.66 ± 5.63_ab_^A^		21.17 ± 1.72_b_^BC^
9	23.85 ± 3.20_b_^A^	20.00 ± 0.00_d_^A^	28.21 ± 9.27_bc_^A^	20.34 ± 0.60_b_^A^	24.61 ± 3.51_cd_^A^	19.35 ± 6.64_c_^A^	23.35 ± 9.56_bc_^A^		29.93 ± 4.63_d_^DE^
12	40.38 ± 4.81_c_^B^	26.92 ± 0.00_c_^AB^	35.36 ± 12.31_cd_^B^	28.92 ± 0.87_c_^A^	30.63 ± 2.85_d_^AB^	32.90 ± 4.06_c_^AB^	31.98 ± 7.20_cd_^AB^		36.86 ± 1.47_c_^B^
24	100.00 ± 0.00_d_^C^	50.77 ± 0.00_d_^B^	50.36 ± 2.86_d_^B^	47.76 ± 3.33_d_^B^	46.86 ± 7.78_e_^B^	42.90 ± 0.54_d_^A^	41.12 ± 6.36_d_^AB^		100.00 ± 0.00_d_^A^

(ii) The percentage of wound closure of NIH 3T3 cell treated with 10% RB maltodextrin (DE 10–14) with 100 ppm of various additives									
0					0.00 ± 0.00_a_^A^				
3	19.23 ± 1.65_b_^C^	15.79 ± 0.00_a_^B^	16.40 ± 0.17_b_^C^	14.81 ± 1.44_b_^BC^	16.33 ± 1.79_b_^E^	17.74 ± 0.00_b_^C^	12.10 ± 0.62_b_^A^	0.00 ± 0.00_a_^A^	
6	32.69 ± 0.70_b_^A^	29.82 ± 2.11_b_^BC^	28.95 ± 4.96_c_^BC^	23.33 ± 6.06_c_^BC^	31.78 ± 0.92_c_^C^	28.09 ± 0.75_c_^AB^	21.66 ± 0.00_c_^A^		
9	48.73 ± 2.32_c_^BC^	42.11 ± 6.88_b_^CD^	36.36 ± 4.73_d_^DE^	30.23 ± 1.52_c_^CD^	38.32 ± 0.78_d_^E^	34.83 ± 0.00_c_^AB^	29.26 ± 0.31_d_^A^		
12	61.13 ± 1.39_d_^D^	52.97 ± 3.68_c_^D^	51.52 ± 2.72_e_^C^	47.78 ± 1.90_d_^C^	47.66 ± 0.75_d_^C^	44.94 ± 4.49_d_^B^	32.98 ± 0.56_e_^A^		
24	100.00 ± 0.00_e_^A^	100.00 ± 0.00_d_^A^	100.00 ± 0.00_f_^A^	100.00 ± 0.00_e_^A^	100.00 ± 0.00_e_^A^	100.00 ± 0.00_e_^A^	100.00 ± 0.00_f_^A^		

**Table:** (b)

Time, h	RB	RBCUR	RBHYD	RBAA	RBARG	RBLA	RBKA	MUL	CTRL
(i) The percentage of wound closure of NIH 3T3 cell treated with 5% RB maltodextrin (DE 10–14) with 100 ppm of various additives									
0					0.00 ± 0.00_a_^A^				
3	3.54 ± 0.76_a_^A^	2.51 ± 3.45_a_^AB^	6.94 ± 3.51_a_^AB^	7.39 ± 1.21_a_^AB^	5.01 ± 0.60_a_^AB^	7.27 ± 1.53_ab_^B^	3.64 ± 2.49_a_^AB^	2.04 ± 0.60_a_^A^	12.04 ± 0.53_b_^D^
6	12.96 ± 4.06_ab_^AB^	7.71 ± 1.06_a_^A^	13.87 ± 3.85_ab_^ABC^	14.43 ± 4.04_ab_^AB^	11.22 ± 4.21_ab_^AB^	20.64 ± 5.11_b_^BC^	10.75 ± 3.00_ab_^AB^	6.89 ± 3.19_b_^A^	21.17 ± 1.72_b_^BC^
9	24.41 ± 5.17_bc_^BC^	21.68 ± 3.48_b_^ABC^	20.65 ± 5.77_bc_^AB^	20.62 ± 2.46_bc_^AB^	23.05 ± 7.83_bc_^ABC^	32.27 ± 7.10_c_^C^	19.58 ± 2.64_bc_^AB^	8.42 ± 2.17_cd_^A^	29.93 ± 4.63_d_^DE^
12	33.50 ± 8.81_c_^AB^	28.14 ± 1.33_b_^A^	27.90 ± 8.70_c_^A^	26.80 ± 6.94_c_^A^	28.86 ± 9.24_c_^A^	44.19 ± 4.17_d_^AB^	27.90 ± 9.47_c_^A^	10.20 ± 0.57_d_^A^	36.86 ± 1.47_c_^B^
24	57.91 ± 5.67_d_^B^	55.56 ± 10.34_c_^B^	53.71 ± 2.41_d_^B^	48.97 ± 6.62_d_^B^	47.90 ± 3.76_d_^B^	46.51 ± 4.05_d_^B^	45.58 ± 8.33_d_^B^	10.71 ± 1.73_d_^A^	100.00 ± 0.00_d_^A^

(ii) The percentage of wound closure of NIH 3T3 cell treated with 10% RB maltodextrin (DE 10–14) with 100 ppm of various additives									
3	19.23 ± 1.65_b_^C^	15.79 ± 0.00_a_^B^	16.40 ± 0.17_b_^C^	14.81 ± 1.44_b_^BC^	16.33 ± 1.79_b_^E^	17.74 ± 0.00_b_^C^	12.10 ± 0.62_b_^A^	0.00 ± 0.00_a_^A^	
6	32.69 ± 0.70_b_^A^	29.82 ± 2.11_b_^BC^	28.95 ± 4.96_c_^BC^	23.33 ± 6.06_c_^BC^	31.78 ± 0.92_c_^C^	28.09 ± 0.75_c_^AB^	21.66 ± 0.00_c_^A^		
9	48.73 ± 2.32_c_^BC^	42.11 ± 6.88_b_^CD^	36.36 ± 4.73_d_^DE^	30.23 ± 1.52_c_^CD^	38.32 ± 0.78_d_^E^	34.83 ± 0.00_c_^AB^	29.26 ± 0.31_d_^A^		
12	61.13 ± 1.39_d_^D^	52.97 ± 3.68_c_^D^	51.52 ± 2.72_e_^C^	47.78 ± 1.90_d_^C^	47.66 ± 0.75_d_^C^	44.94 ± 4.49_d_^B^	32.98 ± 0.56_e_^A^		
24	100.00 ± 0.00_e_^A^	100.00 ± 0.00_d_^A^	100.00 ± 0.00_f_^A^	100.00 ± 0.00_e_^A^	100.00 ± 0.00_e_^A^	100.00 ± 0.00_e_^A^	100.00 ± 0.00_f_^A^		

(iii) The percentage of wound closure of NIH 3T3 cell treated with 20% RB maltodextrin (DE 10–14) with 100 ppm of various additives									
3	7.27 ± 0.00_b_^AB^	11.24 ± 3.08_ab_^A^	11.11 ± 0.00_ab_^A^	10.61 ± 4.77_a_^AB^	12.16 ± 2.66_b_^C^	6.93 ± 1.75_a_^A^	5.32 ± 4.22_b_^A^	0.00 ± 0.00_a_^A^	
6	15.64 ± 3.88_c_^AB^	16.43 ± 0.00_abc_^B^	16.87 ± 3.18_abc_^AB^	22.07 ± 1.92_a_^AB^	18.11 ± 0.24_b_^B^	20.50 ± 7.62_b_^B^	4.98 ± 2.81_b_^A^		
9	20.73 ± 1.47_d_^AB^	20.75 ± 0.00_bc_^B^	23.46 ± 8.67_bc_^AB^	27.37 ± 2.97^AB^	22.43 ± 4.92_b_^AB^	24.65 ± 4.94_bc_^B^	11.63 ± 3.52_b_^A^		
12	22.18 ± 0.84_d_^A^	27.38 ± 3.97_c_^A^	29.22 ± 9.62_c_^A^	33.80 ± 0.00_b_^A^	30.54 ± 3.48_c_^A^	36.01 ± 1.77_cd_^A^	25.58 ± 2.42_c_^A^		
24	72.73 ± 1.69_e_^C^	67.44 ± 0.90_d_^C^	66.26 ± 1.78_d_^C^	49.16 ± 3.76_c_^B^	44.59 ± 4.81_d_^AB^	43.77 ± 3.35_d_^AB^	34.55 ± 2.32_d_^A^		

**Table:** (c)

Time, h	RB	RBCUR	RBHYD	RBAA	RBARG	RBLA	RBKA	MUL	CTRL
(i) The percentage of wound closure of NIH 3T3 cell treated with 10% RB maltodextrin (DE 10–14) with 100 ppm of various additives									
0					0.00 ± 0.00_a_^A^				
3	19.23 ± 1.65_b_^C^	15.79 ± 0.00_a_^B^	16.40 ± 0.17_b_^C^	14.81 ± 1.44_b_^BC^	16.33 ± 1.79_b_^E^	17.74 ± 0.00_b_^C^	12.10 ± 0.62_b_^A^	0.00 ± 0.00_a_^A^	12.04 ± 0.53_b_^D^
6	32.69 ± 0.70_b_^A^	29.82 ± 2.11_b_^BC^	28.95 ± 4.96_c_^BC^	23.33 ± 6.06_c_^BC^	31.78 ± 0.92_c_^C^	28.09 ± 0.75_c_^AB^	21.66 ± 0.00_c_^A^		21.17 ± 1.72_b_^BC^
9	48.73 ± 2.32_c_^BC^	42.11 ± 6.88_b_^CD^	36.36 ± 4.73_d_^DE^	30.23 ± 1.52_c_^CD^	38.32 ± 0.78_d_^E^	34.83 ± 0.00_c_^AB^	29.26 ± 0.31_d_^A^		29.93 ± 4.63_d_^DE^
12	61.13 ± 1.39_d_^D^	52.97 ± 3.68_c_^D^	51.52 ± 2.72_e_^C^	47.78 ± 1.90_d_^C^	47.66 ± 0.75_d_^C^	44.94 ± 4.49_d_^B^	32.98 ± 0.56_e_^A^		36.86 ± 1.47_c_^B^
24	100.00 ± 0.00_e_^A^	100.00 ± 0.00_d_^A^	100.00 ± 0.00_f_^A^	100.00 ± 0.00_e_^A^	100.00 ± 0.00_e_^A^	100.00 ± 0.00_e_^A^	100.00 ± 0.00_f_^A^		100.00 ± 0.00_d_^A^

(ii) The percentage of wound closure of NIH 3T3 cell treated with 10% RB maltodextrin (DE 15–19) with 100 ppm of various additives									
3	7.22 ± 1.54_ab_^AB^	10.96 ± 0.00_a_^AB^	11.41 ± 0.00_b_^BC^	9.29 ± 2.68_b_^ABC^	16.29 ± 3.23_a_^AB^	7.02 ± 4.49_b_^AB^	2.93 ± 0.62_b_^A^	0.00 ± 0.00_a_^A^	
6	16.58 ± 2.07_c_^A^	18.36 ± 0.00_a_^A^	21.75 ± 3.75_c_^BC^	25.70 ± 0.00_c_^AB^	29.14 ± 4.80_b_^C^	18.73 ± 0.75_c_^B^	13.20 ± 0.00_c_^A^		
9	22.99 ± 6.95_d_^BC^	26.85 ± 9.73_b_^ABC^	33.42 ± 1.76_d_^BC^	32.51 ± 1.52_d_^BC^	39.71 ± 5.30_bc_^C^	20.40 ± 0.75_c_^AB^	17.30 ± 0.00_d_^A^		
12	56.28 ± 3.40_d_^BC^	50.41 ± 7.69_c_^D^	49.36 ± 5.38_e_^CD^	45.82 ± 1.80_e_^CD^	45.14 ± 8.24_c_^CD^	31.10 ± 0.00_d_^AB^	20.23 ± 0.00_e_^A^		
24	100.00 ± 0.00_e_^A^	100.00 ± 0.00_d_^A^	100.00 ± 0.00_f_^A^	100.00 ± 0.00_f_^A^	100.00 ± 0.00_d_^A^	46.48 ± 0.00_e_^B^	28.15 ± 0.00_f_^C^		

(iii) The percentage of wound closure of NIH 3T3 cell treated with 10% RB maltodextrin (DE 20–24) with 100 ppm of various additives									
3	13.56 ± 3.34_b_^C^	9.30 ± 0.00_b_^C^	12.33 ± 0.00_b_^C^	17.11 ± 0.00_b_^A^	8.45 ± 0.00_b_^A^	14.10 ± 0.00_b_^D^	8.62 ± 0.00_b_^AB^	0.00 ± 0.00_a_^A^	
6	23.73 ± 0.00_c_^AB^	23.26 ± 6.91_b_^AB^	24.66 ± 0.00_c_^AB^	27.63 ± 0.00_c_^A^	25.35 ± 0.00_c_^AB^	26.92 ± 0.00_c_^B^	22.41 ± 0.00_c_^AB^		
9	33.90 ± 0.00_d_^CD^	35.47 ± 4.24_c_^BC^	41.10 ± 0.00_d_^E^	35.53 ± 0.00_d_^AB^	25.35 ± 0.00_c_^A^	37.18 ± 0.00_d_^D^	41.38 ± 0.00_d_^E^		
12	47.46 ± 0.00_e_^DE^	47.67 ± 4.71_c_^DE^	49.32 ± 0.00_e_^E^	44.74 ± 0.00_e_^BC^	35.21 ± 0.00_d_^A^	46.15 ± 0.00_e_^CD^	53.45 ± 0.00_e_^F^		
24	66.67 ± 0.00_f_^D^	61.63 ± 3.46_d_^BC^	65.75 ± 0.00_f_^D^	59.21 ± 0.00_f_^A^	47.89 ± 0.00_e_^A^	57.69 ± 0.00_f_^B^	63.79 ± 0.00_f_^CD^		

^a^ Each data was expressed as mean ± standard deviation of triplicate determinations. Mean values with different superscripts in the same column are significantly different *p* < 0.01. Mean values with different superscripts in the same row are significantly different *p* < 0.01.

RB: broken rice maltodextrin; CUR: curcumin; HYD: hydroxyproline; AA: ascorbic acid; ARG: L-arginine; LA: lactic acid; KA: kojic acid; MUL: Multidex; CTRL: control.

**Table 2:** *In vitro* wound scratch assay on NIH 3T3 fibroblast cell using 10% RB maltodextrin DE 10–14 and 10% Multidex (commercial wound dressing).

Time, h	Broken rice maltodextrin DE 10–14	Multidex (commercial wound dressing)
0	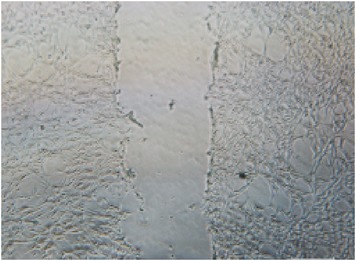	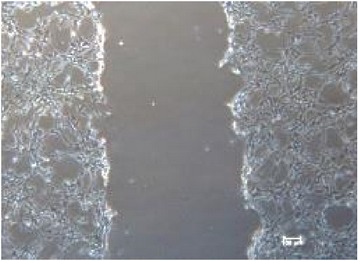

6	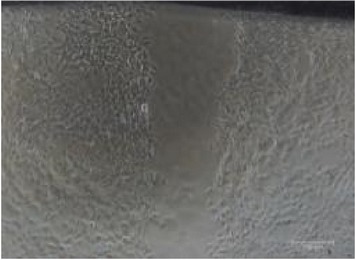	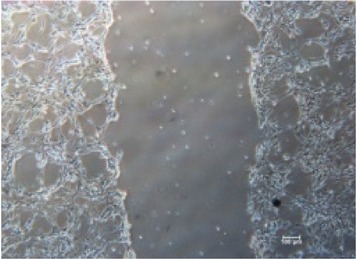

12	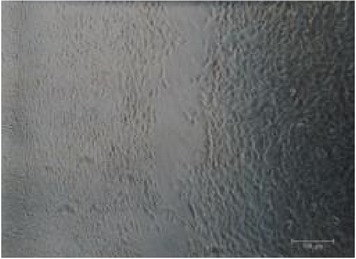	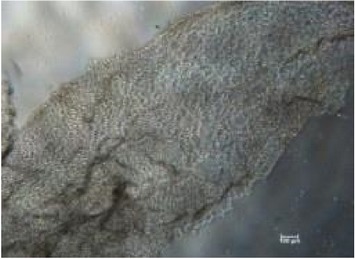

24